# The Work Undertaken by Patients and Their Families Whilst in the Emergency Department

**DOI:** 10.1111/hex.70602

**Published:** 2026-02-16

**Authors:** Michael Clancy, Mark J. Johnson, Joanne Turnbull

**Affiliations:** ^1^ School of Health Sciences University of Southampton, Hampshire UK; ^2^ School of Human Development and Health, Faculty of Medicine University of Southampton Southampton Hampshire UK

## Abstract

**Introduction:**

Patients sometimes stay a considerable length of time within Emergency Departments (EDs) and often leave with an incomplete understanding of their health problems and treatment. The complexity of how patients and their families make sense of emergency care and what they do throughout their journey is incompletely described, typically relying on retrospective interviews. This study describes the development of a typology of their thinking and doing, and how it can be used to explain reported outcomes and potentially make the patient's journey easier.

**Method:**

This paper draws on the concept of patient work to explain how patient diagnostic and treatment journeys relate to the work of staff and their endurance of that journey. Observations and informal interviews with 51 adult patients and 8 family members were undertaken in an English ED. We construct a typology to develop the concept of patient and family work during their ED journey, and how this is shaped by a healthcare context (e.g., limited patient–staff relationships and overcrowding) that is very different from chronic illness contexts that previously applied the concept.

**Results:**

Findings demonstrate how patients' sensemaking work is shaped by their expectations and informational exchanges with the clinician, but gaps in sensemaking exist. Sensemaking interacts with endurance work, that is, long waits in a noisy, public environment. Families participate in and often ameliorate patient sensemaking by seeking clarification, providing information, meeting patients' commitments and easing endurance work.

**Conclusion:**

Collective analysis of patients' sensemaking and endurance work demonstrates how their diagnostic and treatment journeys shape patient experiences and navigation of ED healthcare encounters, with the potential to inform interventions to ease this work.

**Patient and Staff Contribution:**

This study was informed by prior co‐design and observation work with patients and staff during the development of a checklist to help meet patient information needs. This highlighted the centrality of the patient–staff interaction and the need to better understand it by directly observing the patient journey.

**Abbreviations**
DTFU plan: diagnostic, treatment and follow‐up plan
ED: Emergency Department

## 1 Introduction

### 1.1 Background

The care delivered by NHS Emergency Departments (EDs) is under severe pressure from overcrowding and long waits [1, 2]. This leads not only to worsening patient experience [3, 4] but also to increased morbidity and mortality [5]. When a process of care is not working well, understanding the role of the patient and their family and staff during their journey within the ED is crucial if we are to understand what is happening and why these outcomes are at their current levels and, importantly, how it can be improved.

Interventions have, however, focused on improving the flow of patients through the ED [6, 7]. There is less focus on the patient, their family and their role in those processes within the ED and how those roles could be changed to be more effective and in turn, potentially less resource‐intensive.

### 1.2 Previous Research

What patients experience whilst in the ED has been well described by extensive qualitative research and it has been comprehensively reviewed [8, 9]. However, the majority of these papers used semi‐structured interviews, usually conducted well after the event. They provide extensive descriptions by patients of the staff, what they did and how the patients felt. They also report what waiting felt like and identified the features of poor ED environments. There was less focus on what patients said or thought when interacting with staff, and explanations for the outcomes of the patient experience were infrequent.

Observational studies are infrequent, often studying only part of the ED journey. By producing descriptions and explanations based on the way in which people actually behave [10], they have the potential to provide a fuller, more nuanced understanding, describing the roles of the patient, staff and family and what happened. The work by Hillman [11] provides an excellent theorisation of the process of legitimation by the patient during their interaction with staff within the triage process.

### 1.3 The Concept of Work and Use of the Medical Research Council Framework

Patient experience is the product of the interaction between staff, patients and family within the context of the ED. If we are to explore and understand the underlying thinking and doing of patients and families, we need a means of separating their experience into its component parts. At the same time, we need a framework that will enable such data to be summarised and facilitate exploration of relationships between the data.

Sociological concepts of illness experiences relating to diagnostic and treatment journeys often tend to focus on people experiencing an illness over a period of time. One such body of work is the sociology of patient illness work, which describes the concept of patient illness work ‘as anything the patient does in relation their own healthcare’ [12, 13]. Strauss et al. [14] used this to recognise the role of patients and their families and to separate that work from that of healthcare staff. They described the work of patients both in the setting of chronic illness and within the hospital, and decomposed that work into a number of work streams (cognitive, physical, social, behavioural and articular work) that were both visible and invisible to staff. Such work was then related to illness, its treatment and its social consequences [15]. This concept places focus on the role of the patient and family by providing a means of revealing and understanding the lived experience of patients in managing their healthcare and navigating the healthcare system. It recognises that patients and family have agency and are actively engaged in various forms of work rather than being passive participants [16]. Describing that work and how it relates to the work of staff potentially provides explanations of the outcomes of this patient journey [14]. It also provides an alternative lens for staff to view the patient not just from the medical aspects of illness and service processes but also the emotional, social and logistical challenges that the patient faces. Such an approach has been successfully developed to describe patient and family work in a variety of chronic illnesses (e.g., [17]). This led to the measurement of that work [18] and to explanatory models of burden of treatment [19, 20]. Interventions that recognise and support patients in their work rather than adding to their burden have benefited patients in terms of reduced work, and improved patient outcomes and experience, for example, when patients transition from the hospital to the community [21]. Yet, concepts of patient work are not commonly applied to emergency care contexts. Where they exist, they have focused on help‐seeking and access to emergency care rather than the diagnosis and treatment journey within the ED (e.g., [22]).

If such work was described, exploring how that work can be improved (made more effective, efficient and cheaper) by staff working differently with the patient and family in a setting that is considered difficult to deliver patient‐centred care^23^] would be important.

A framework that enables patient and family work to be placed alongside that of staff with its outcomes, and can accommodate different stages of the patient journey within the context of the ED, can be used to help structure data analysis and facilitate theorisation about the processes of care that patients and families experience.

The Medical Research Council (MRC) guidelines on the process evaluation of complex interventions [24] present a detailed framework for the evaluation of the process of care delivered building on earlier guidance [25] around implementation, mechanisms and context. It was designed to unpack how and why interventions work or do not work in real‐world settings and to aid both evaluation and theorisation. We describe the features of the model and how we apply it to the ED journey. By considering the current care provided by staff to patients as the intervention, we use it to enable a deeper understanding and theorisation of the processes involved in the delivery of ED healthcare using qualitative data from the empirical fieldwork. Later in the discussion, we combine this with national survey outcome data and use the framework to theorise how patient work could be made easier. We do not use the framework in the more conventional way, which is to conduct an evaluation of a novel intervention, which also requires additional quantitative methods and outcome data.

The MRC (2015) model consists of the following components:

(1) A logic model which can be considered a description of the structures in place to deliver an intervention, the intended activities and intended outcomes. It provides a description of the causal assumptions, that is, what happens and why. (2) The intervention: that which is delivered by staff to patients with the aim of improving their health. (3) Implementation describes how delivery is achieved and what is delivered. (4) The mechanism of impact describes the patient's responses to and interactions with the intervention, describing both expected and unexpected pathways and consequences and why patients do or do not engage as planned and explain the outcomes. (5) Outcomes, both intended and unintended. (6) The context is defined as anything external to the intervention that impedes or strengthens the effect of the intervention (and includes circumstances, organisational resources and norms and attitudes).

We apply this framework to the ED journey: (1) The logic model consists of a series of ED staff patient interactions designed to assess the patient, leading to symptom relief, and a diagnosis and treatment plan with the patient waiting between these encounters. (2) The intervention: the care provided by staff to the patient and their families. (3) How this is implemented by staff, that is, how and what is delivered, and we call staff work. (4) The mechanism of impact, that is, how the patients and families respond and interact with staff and the ED setting, and we call patient and family work. (5) The outcomes of these interactions, for example, patient understanding of their diagnosis and treatment. (6) This happens within a context, for example, overcrowding, or a noisy environment.

By combining the concept of work with the MRC approach, a simple framework can be assembled (Table 1). This describes the patient journey in stages, with a series of work themes, each with staff, patient and family work leading to outcomes that occur within the department and that influence what happens beyond it.

**Table 1:** Analytic framework integrating the concepts of work and process evaluation.

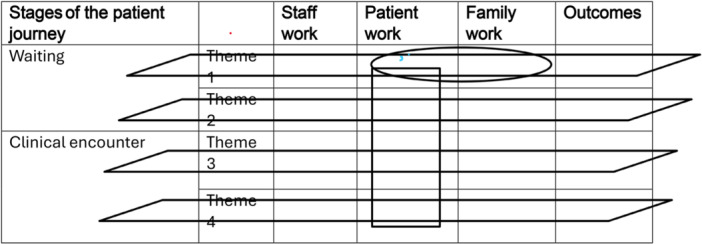

The rows represent work themes, that is, the different types of work that the patients undertake within the stages and how that relates to what the staff and the family are doing. Within each specific theme, there is an opportunity to focus on the individual cells and consideration of how that work can be improved and how the cells are inter‐related, for example, how the work of the family and the patient interacts (the oval in Table 1). It is also possible to gain a sense of the totality of work undertaken by each group (the rectangle in Table 1).

This model provides a detailed framework focused on the evaluation of a complex process, identifying the important components and their interactions. It closely aligns with the aims of this study. The later revised framework [26] is much broader, concerned with the evaluation of the complete life cycle of an intervention (development, feasibility, evaluation and implementation), and was less well suited to the narrow focus of this study.

This paper aims to apply the concept of patient and family work to the ED patient journey and develop a typology of work in the ED context. This indicates that the ED contextual factors (diagnostic uncertainty, limited patient–staff relationships, overcrowding and short time frame) are very different to contexts to which the concept has been previously applied (e.g., chronic illness and end‐of‐life care).

### 1.4 Study Aim

The aim of this study is to describe patient and family work during their ED journey and explain how this is shaped by the ED context. It aims to examine how this relates to the work of staff, explain the outcomes of that work and, by characterising such work, explore how patient work can be made easier.

## 2 Methods

### 2.1 Setting

The setting for this study was a Type 1 ED (i.e., consultant‐led and open 24/7 with full resuscitation facilities) within a university teaching hospital that served both an urban and rural population of approximately 500,000, with an annual attendance of 120,000 patients.

### 2.2 Fieldwork

An ethnographic approach was used that included observation, informal interviews (conversations) and documentary review. Observation was conducted by the field researcher (M.C.) accompanying the patient throughout their journey. Brief informal interviews [27] were conducted to explore the patients' understanding by asking about their expectations, worries, concerns and their views on waiting and privacy. Notes were taken in the field contemporaneously and written up fully, typically 1–2 h later, along with reflexive thoughts. The patients' ED notes, which included prehospital care notes, were reviewed > 24 h later to explore the clinicians' perspective, that is, what they chose to record and how they recorded it. Discrepancies between the field notes and the clinical record were assessed.

The COVID pandemic occurred after recruitment had started and had to be suspended in March 2020 when a stay‐at‐home order was mandated in the United Kingdom, referred to as ‘lockdown’. By this time, 15 patients and 8 relatives had been recruited. When recruitment was restarted (September 2020), the process of care delivery within the ED had significantly changed. Waiting rooms were no longer in use, triage was conducted outside of the department and family and friends were no longer permitted to attend. This restricted the stages of the ED journey that could be observed to the clinical encounter. The exclusion of family and friends meant that the role of the family within the ED could no longer be observed but was revealed by the content of the phone calls between the patient, staff and family members, which was shared with the researcher (M.C.), showing the work that they were engaged in whilst outside of the ED.

### 2.3 Recruitment

Adult patients, who were not being treated in a resuscitation area or had a mental illness or learning disabilityere approached for recruitment. Participants were purposefully selected to support a theoretical generalisation [10, 28]. The following factors were taken into account during recruitment:

1. The age of the patient.
2. The time of the day and day of the week.
3. The patients' presenting complaint (as reported at the time the patient registered). This was used to identify which conditions could be expected to be straightforward (e.g., lacerations and sprains), complex (e.g., patients with multiple comorbidities) and uncertain, with a wide range of possible diagnoses (e.g., chest pain, headache and abdominal pain). The presenting complaint was also used to gauge their potential severity (in terms of potential disruption to life and need for admission).
4. Which clinician saw the patient, as it would be expected that the patient and family work related to the interaction with staff could be influenced by their grade and training.

Patients provided written consent, including permission to review their ED notes for this attendance.

### 2.4 Analysis

The analysis identified themes from the data through the process of coding, as described by Braun and Clarke [29]. This involved identifying meaningful segments and assigning names, then combining codes into broader categories or themes, identifying patterns and making comparisons in order to describe what was happening. The MRC framework facilitated analysis by displaying the different work themes and the roles of staff, patients and families, aiding exploration of relationships between the data. The different data sources (field notes, audio recordings of informal interviews and documents) were brought together to provide a more complete understanding of the patient journey and context. The researcher undertaking the fieldwork (M.C.) was an experienced emergency physician who was overt in their role as researcher and disclosed their past clinical role to both staff and patients. The benefits of being such an ‘insider’ included a greater understanding of what was happening, an awareness of the flow of the interactions and greater intimacy that promoted truth‐telling [30]. However, such expertise and familiarity risked failing to see the obvious rather than seeing things anew. This was anticipated and protected against by the use of reflexive writing, and using summaries of such thinking throughout the project to facilitate the sharing of data, thoughts and analysis with others to enable peer debriefing. M.C. led the coding of the data, but emerging codes were discussed with the wider team (J.T. and M.J.J.). This formed the basis for a coding scheme, which was refined and applied to all transcripts. In the later stages, a typology was created to map interpretations and to make relationships and connections, referring back to the coding and thematic analysis throughout this process.

### 2.5 Ethical Approval

Ethical approval was provided for this study by the NHS Health Research Authority and approved by the hospital research unit and the University. Participants were informed that all information would be handled confidentially, anonymised and any identifiable data would be removed before being used to produce research findings and reports.

## 3 Results

### 3.1 Quantitative Description of the Cohort Studied

One hundred hours were spent in the field recruiting and observing 51 patients' ED journeys between September 2019 and November 2020. The patients' age and sex, day of attendance, time of day of attendance, the level of ED activity, their clinical journey and the grade of staff who saw the patient and if they attended before or during the COVID pandemic are reported in Table 2.

**Table 2:** Description of patients' sex, age, day and time of attendance, level of activity, clinical journey and grade of staff who saw the patient.

Characteristic	Categories	Result *n* (%)
Sex	Male Female	29 (57) 22 (43)
Age	< 29 years 30–49 years 50–69 years > 70 years	13 (25) 16 (31) 12 (24) 10 (20)
Day of attendance	Attended during a weekday Attended at the weekend	38 (69) 13 (31)
Time of day	0600–1200 1200–1800 1800–2400	15 (29) 28 (55) 8 (16)
Level of activity	It was recorded as busy when patients were queuing within the clinical areas because all the cubicles were full	13 (25)
Clinical journey	Patients admitted or discharged from the ED Patients transferred to the Clinical Decision Unit Referred for a specialist opinion	31 (61) 5 (10) 15 (29)
Grade of staff	Patients seen by junior medical staff	18 (35)
	Patients seen by advanced nurse practitioners	18 (35)
	Patients seen by senior medical staff	15 (30)
COVID pandemic	Recruited before first COVID lockdown Recruited after the first COVID lockdown and during the pandemic	15 (29) 36 (71)

The presenting symptoms were typical of ED practice: limb pain/injury (16), abdominal pain (8), chest pain (7), ear, nose and throat (2), dental pain (2), back pain (2), breathlessness (2), loss of consciousness (2) and others (9) such as rectal bleeding and palpitations.

### 3.2 Qualitative Findings

The findings present nine patient work themes, which are grouped into the two overarching themes of patient sensemaking and patient endurance work. Four family work themes are also presented. The summary table (Table 3) shows a typology of such work and provides an early overview of the findings, which are then discussed. These themes are supported by field note excerpts (highlighted), which avoid the use of quotation marks, as permission to use verbatim quotes was not explicitly sought from the participants.

**Table 3:** A typology of patient and family work.

Overarching patient work themes	Patient work themes	Family work themes
Patient sensemaking	Expectations	Information provider
	Understanding waiting	Clarifier and enquirer
	Information provider	Organiser
	Information receiver	Provider of basic needs and comforter
	‘Keeping the show on the road’	
Endurance	Waiting	
	Basic needs	
	Noise and lack of privacy	
	Discomfort of symptoms and procedures	

### 3.3 Patient Sensemaking Work

Patient sensemaking work encompasses the cognitive effort expended by patients to understand their health problem and its treatment, and the associated care processes of the ED. Patients are engaged in the continuous gathering and exchange of information in order to update their understanding and to be able to make decisions [31].

#### 3.3.1 Arrival at the ED: The Work of Expectation

By the time the patient had arrived at the ED, their sensemaking about what to expect, which was routinely asked by the researcher, was typically well developed. This was informed by their prior experience and by contact with others, specifically healthcare providers such as NHS 111 (a 24‐h urgent care telephone line that addresses and signposts callers to available services). Patients often had clear ideas about what tests they might need and possible diagnoses and treatments. They used their prior experience to legitimate and inform clear expectations of what the patient wanted to happen this time.

Patient 4 was a female in her early 20s with a severe sore throat and pyrexia, who had attended recently with the same symptoms, but who judged that the treatment on that occasion had been delayed. She reported to the researcher that she wanted to get it sorted out, as every part of her body hurt. She stated that previously, she had required intravenous antibiotics, which had been delayed. She wanted to be taken seriously. When the researcher asked what she expected to happen next, she said she expected to have tests and receive antibiotics, and to be admitted.

#### 3.3.2 Waiting: The Work of Understanding Waiting

The wait to see the clinician was the longest part of the journey, and patients uncomplainingly endured the uncertainty caused by the practice of staff not providing waiting time estimates. Although they wanted to know, they did not ask. Even in the absence of this information, they still needed to decide what to do. They made sense of this uncertainty by feeling that they had to remain in the waiting area to avoid missing their turn and that they could not use that time differently (e.g., go for a drink, and go and make calls in areas with better mobile reception).

#### 3.3.3 Clinical Encounter: The Work of the Patient as Information Provider and Receiver

During the encounter with staff, it was evident that in all cases, patients had two separate sensemaking tasks: First, to act as an information provider to the clinician's enquiry whilst they were being interviewed and examined and second, to act as the information receiver taking in the clinician's diagnosis, treatment and follow‐up plan (DTFU plan). The clinician led the enquiry, determining what information was sought from the patient.

Patient 13 was a male in his late 50s with a syncopal episode following his discharge after an operation as a day case. The doctor asked what had happened, and the patient replied that he had more or less fainted. The doctor asked what happened after that, and the patient's wife reported that the patient lost consciousness and was taken from the car park to a nearby building by passing healthcare workers. The patient reported that he had his blood pressure and temperature taken and that he felt quite clammy. The doctor then asked if the surgery was under a local anaesthetic and whether the patient had fasted before the operation. The patient replied that the surgery was carried out under a local anaesthetic, and that he had fasted. The doctor then asked how the patient was currently feeling, and the patient stated that he felt normal. The doctor went on to establish the patient's past medical history and the medications that he was currently taking. The patient's wife showed a prescription to the doctor. The doctor then asked the patient about who he lives with and his job. The doctor then went on to enquire about previous episodes of syncope (loss of consciousness) and explored the associated symptoms of chest pain and breathlessness. He asked if the patient had travelled abroad recently (because of the association of air travel with venous thrombosis, which can cause syncope).

In this case, the doctor led the enquiry and followed the conventional history‐taking template of questions. At no stage was the patient or the patient's wife asked their opinion as to what was important to them, what they might expect or what their principal concern was. The patient did not contribute other than to answer the doctor's questions. In this case, the wife was an important provider of information.

Although staff often started with open questions, these quickly led to closed questioning focusing on the clinical problem. Patients' participation was usually limited to answering the clinician's questions and rarely stepped outside of the clinician's enquiry. Occasionally, they asked questions for clarification and volunteered information spontaneously. The exploration of the patients' expectations, views, concerns and what was important to them was very infrequently undertaken by staff or shared by the patient.

However, when patients felt that they were not being taken seriously, which was infrequent, they emphasised their symptoms and concerns by repetition.

Patient 32 was a woman in her late 40s with breathlessness, chest pain and a slow heart rate. The patient reported to the researcher that she could not carry on feeling short of breath just standing at her sink, and that she expected more tests, and possibly admission to the hospital or an urgent outpatient appointment. The doctor arrived and asked to go over the patient's story. The patient established early on that she was breathless while doing basic activities and that she felt like she was blacking out. She then expressed concern that her symptoms might be related to a COVID infection or that her diabetes might be affecting the nerves that controlled her heart. She went on to reiterate that she cannot carry out general activities and repeated her concern about COVID and diabetes as a cause. She repeated on two further occasions that she cannot do the washing up and was breathless while carrying out basic activities. She made it evident to the doctor that clearly something was not right. She subsequently underwent further testing and was seen by a specialist that night.

This experienced patient had her own theories as to why she was unwell, reflecting her sensemaking up until that point. She reiterated her symptoms and concerns repeatedly. She worked to make her case, as she was concerned that she would be sent home without a diagnosis.

When clinicians provided information to the patient (i.e., when the patients were information receivers), in all observed cases, there was consistent delivery by the clinician of the (DTFU) plan. There was infrequent coverage of the implications of what was proposed in terms of what this meant for the patients, for example, their employment, ability to drive, self‐care and care of dependants. This apparent non‐coverage by the staff may have resulted from both time pressure and assumptions made by staff, for example, that another service would explain, that the patient already understood or that help would be available and the patient would manage.

Patient 12 was a woman in her early 70s with a shoulder fracture; her husband was with her. The doctor confirmed that the patient's nerves and artery in her arm were okay, and no urgent intervention was needed (diagnosis). She indicated that the patient would need a collar and cuff to take the weight off (treatment) and a fracture clinic follow‐up in 1 week's time (follow‐up). She reiterated that an operation was not needed and that the orthopaedic department would review her. She was advised to seek advice (follow‐up) if she developed any weakness or became unwell. She was advised to rest, take fluids and get on top of the pain (treatment). The doctor asked if what she proposed was acceptable and if the patient had any questions. The patient replied that she did not.

In this case, although the DTFU plan was well covered, there was no discussion about how long it would take to recover, how the patient would manage with activities of daily living such as dressing and going to the toilet or when she could drive, all potentially significant implications for the patient.

The staff's enquiry of the patients' understanding of the DTFU plan was most commonly by asking the patient if they had ‘any questions’, as in this case, and rarely was the patients' understanding explicitly enquired of. There was no evidence of staff requesting patients to explain their understanding of what was proposed using their own words (‘playback’). Patients responded infrequently after the delivery of the DTFU plan, seeking clarification of what was proposed and rarely asked about its implications. Checking by staff of what patients understood, that is, the patients' sensemaking before discharge of what was proposed, was missing. This meant that staff did not know what sense the patient had made of what was proposed. This was not effectively addressed by staff asking if the patient had ‘any questions’, as this did not assess the patients' comprehension of what was proposed. It relied on the patient to identify what s/he did not know (known unknown), ignored what s/he did not know they know (unknown unknowns) and assumed that if questions are not asked that the patient understood.

### 3.4 The Work of ‘Keeping the Show on the Road’

Outside of the immediate clinical problem, patients were engaged in making sense of their current situation and its impact on their lives to determine the actions needed to meet their obligations to their dependants and their workplace, by organising, contacting and informing, that is, ‘keeping the show on the road’.

Patient 3 was a female in her early 70s with hip pain and a dependant husband. She took a call from her daughter, who was abroad on holiday. The daughter asked if she should come home, and the patient said no. The patient phoned her husband to let him know she was ok and later called her granddaughter to request her to check on her husband (who had mild dementia).

Concurrent with this patient making sense of their clinical problem, she still had work to do. She had to organise care for her husband, to inform and reassure her daughter and to make a decision on whether her daughter should return from holiday or not.

### 3.5 Patient Endurance Work

#### 3.5.1 The Work of Discomfort, Waiting, Noise and Lack of Privacy and Meeting Basic Needs

When patients have unpleasant experiences, this may be considered as endurance work. Patients suffered from physical symptoms (e.g., pain and the discomfort of procedures) and waited for prolonged periods whilst being cared for in noisy departments. Patients occasionally became hungry, thirsty and cold, and were usually helped by their families. Any uncertainty about the significance of their symptoms remained unresolved whilst they waited. This had to be endured. Their sensemaking about their condition was put on hold. They continued to worry, more so the longer they waited.

Patient 13 was a male in his early 50s with syncope. The patient reported that when he first came in, he was not worried, but after waiting for the doctor, the unknown (the cause of his collapse and if there was something wrong) started to worry him and also worried about what was going to happen. The patient's wife also reported that waiting for the outcome of the consultation was the worst part, and that waiting felt like an eternity.

Patients seen in this busy ED with cubicles separated by curtains often had their privacy compromised, with others able to hear their conversations and they in turn were able to hear conversations of others. Privacy was important to patients, and they wished on occasion that there could be more privacy. Loss of privacy, despite being accepted, was something that had to be endured.

### 3.6 The Supportive Role of the Family

The patient's family played important roles. They provided information, enquired, clarified and acted as the patient's advocate, in addition to thinking of and meeting the patient's commitments beyond the department. They acted to bridge communication gaps with the clinician and beyond the ED (with the patients' dependents, and their employers), aiding the patients' sensemaking work. They also worked to reduce the work of endurance, especially whilst waiting, by providing emotional support and physical comfort.

#### 3.6.1 Family as Clarifier and Enquirer

Here, the mother sought to clarify where the patient was in their journey, and to confirm on the patient's behalf that she was going to be admitted.

Patient 4 was a single mother with two young children expecting admission to the hospital. When the patient's mother arrived, she asked her daughter about the journey to that point and asked her if the staff had administered antibiotics yet and later asked the doctor if the patient would be staying in.

#### 3.6.2 Family as Information Provider/Information Keeper

Patient 13 had collapsed and lost consciousness in the presence of his wife. He informed the doctor that he would have to ask his wife what had happened, as he did not know, and she went on to describe his collapse. She also took responsibility for looking after his medical documentation and showed his prescription to the doctor.

#### 3.6.3 Family as Organiser and Working With the Patient to Meet Concomitant Obligations

Patient 15 was a 65‐year‐old woman with abdominal pain; she had been informed that she was to be admitted. Her daughter then undertook the task of informing friends, and work, and to bring clothes for the patient, whilst her husband took up the task of feeding the cats, and sorting out work and the car (left at work) the following day.

#### 3.6.4 Family as Provider of Basic Needs and Comforter

Family members augmented what was provided by staff in meeting basic needs of the patient, bfor example, providing food and drink, clothing and making the patient more comfortable. Family members also shared in the waiting and wanted to comfort the patient in relation to their clinical problem.

## 4 Discussion

The fieldwork findings showed that not all the potentially relevant information for diagnosis and treatment may be obtained by the clinician and that the patient may not understand or have all the information that they need. We discuss how this relates to the wider literature, the findings of national surveys and how the work that families undertake can help. We then discuss how the work of the patient might be made easier.

### 4.1 Informational Gaps‐Incomplete Sensemaking

What information the patient provides whilst undertaking the work of information provision is determined by the enquiring clinician, who is focused on the immediate clinical problem [32, 33]. They do not explore the patients' views; this has been reported elsewhere [34]. Patients respond to the clinician's questions rather than relating their own story or questioning the clinician [35, 36].

This incomplete information exchange can lead to key information relevant to the diagnosis and treatment being missed and the patient's perspective being unappreciated [36, 37]. This may be considered a gap in the clinician's sensemaking, which is needed to develop the diagnosis and treatment plan. This lack of enquiry into the patients' point of view is reflected by NHS ED surveys, which found that only 61% had definitely enough time to discuss their condition [4], and for patients with anxieties or fears, these were not discussed with their clinician 25% of the time. The existence of this gap in clinician sensemaking is supported by estimates of diagnostic error within EDs of the order of 10%–15% [38], much of which is thought to relate to the bedside interaction between the clinician and the patient [39].

During the information‐receiving work of patients, they may not understand what was said and may be provided with incomplete information. This second informational gap may then remain undetected because the patients' comprehension is not assessed, and patients may not ask comprehension‐checking questions themselves. These features of patients' information‐receiving work are identified in the literature [35, 40, 41]. That this gap exists is supported by the NHS data [3, 4], where only 69% stated that their condition was explained completely and 30% of patients stated that the information was not understandable, or was understandable to some extent. It was also often incomplete; for example, less than half were told when they could return to work or drive, and 33% did not have sufficient information to help care for their condition at home. Such gaps could have major consequences for health outcomes, given that compliance with the treatment and proposed follow‐up is dependent on the completeness of the information and the patient's understanding of it [42].

This empiric work confirmed that busy staff choose to focus on the immediate clinical problem and its solution at the expense of relational work and the work of checking comprehension. Such dialectic tensions have been previously noted [32]. The patients' compliance and non‐enquiry may reflect their dependant position (i.e., they have an emergency that they cannot solve themselves) within a busy system. This may be another example of what Pilnick [43] refers to as ‘bad practice but good care’, that is, clinicians were unable to deliver patient‐centred care, but delivered the best care that they could provide, given the service circumstances.

### 4.2 Families Bridging the Gap

Families often play a pivotal role in bridging the gap in the clinicians' sensemaking that follows their sometimes incomplete enquiry. By providing missing information, seeking clarification and presenting the patient's point of view, concerns and expectations, they compensated for the incomplete delivery of information both to and from the patient. Similarly, the patient's sensemaking of the DTFU plan may be incomplete due to the failure of the clinician to deliver all the important information and check that the patient understands what has been said. Families bridged that gap by checking their understanding and that of the patients, and by using their knowledge of the patient and their circumstances to check that the DTFU plan is appropriate. During the waiting periods, families helped to ‘keep the show on the road’ by working with the patient to meet their ongoing commitments to others (family/employer/animals). They shared the sensemaking work needed to understand the implications of the ED visit. Alongside this, families helped with the patient's endurance work by ensuring that their basic needs were met and that they were comfortable.

The work themes reported here are consistent with previous reports of patient experience [8, 9, 44], including a recent study from an overcrowded English ED [45], and what patients report as being important to them after they have left the ED [46].

### 4.3 Making Patient Work Easier

By dividing the patient experience into work themes and their component parts, this study prioritises the patient perspective. It invites the identification of solutions as to how to make their work easier or more effective by what patients, family and staff may do differently. By understanding the work that patients are doing, staff are then in a position to adjust their practice to positively impact that work and become more patient‐centric.

For example, if we revisit the theme of the patient as information receiver, Row 1 of Table 4 describes the findings from this empiric study. First, there was incomplete delivery of information that could be expected to impact health outcomes. Second, the patients' understanding of their DTFU plan was rarely enquired of. Row 2 addresses the issue of incomplete information delivery by suggesting that staff information delivery adheres to existing guidelines as to what should be covered [47] and use of a checklist. This also requires the patient to take in and be able to recall this additional information, increasing their sensemaking work. Row 3 suggests comprehension checking by staff of the patients' sensemaking by having patients explain in their own words their understanding [49] of the DTFU plan and the clinician correcting any misunderstanding and filling in missing information.

**Table 4:** Interventions to improve the work of patients as information receivers.

Theme	Staff work	Patient work	Family work	Outcome
1. The patient as information receiver.	Consistent delivery of DTFU. Infrequent coverage of implications. Patient comprehension most commonly assessed by asking ‘any questions?’ Patient understanding rarely enquired.	Patients infrequently asked questions or challenged the clinician.	Clarifier/enquirer	There is uncertainty as to the patient's comprehension of the DTFU plan and its implications.
2. The provision of important information aided by a checklist.	Staff provide the recommended information [47] using a checklist.	Patients take in the information and are able to recall it.		Patients have the information needed for their care beyond the ED.
3. Checking of comprehension using the playback technique.	Staff take time to enquire as to patients' understanding in their own words [48].	Patients ‘play back’ their understanding of what the clinician has proposed [49].		Improved compliance and better health outcomes.

Extending such an approach across the other sensemaking themes, it is possible to identify examples of how staff, by acting differently, could potentially impact the work that patients undertake [50].

### 4.4 Limitations

1. Although COVID disrupted both the recruitment and the care processes, and observation of the family was not possible, the nature of the work undertaken by patients within the clinical encounter was not obviously different.
2. Using semi‐structured interviews immediately after the end of the journey would have furthered the understanding by exploring what patients and family had thought and done during that journey, and the recall of details would have been fresh.
3. It is possible that the field researcher impacted the behaviour of staff (by virtue of being a researcher and previously a clinician in the same department). It was evident after prolonged observation that staff behaviour was consistent, suggesting minimal impact by the researcher. Patients were informed that the field researcher was previously a clinician in the department, and no patient asked the researcher any questions of a clinical nature.
4. A single‐centre study like this one limits the empiric generalisabilty of these findings. We sought, instead, by purposeful sampling from a rich case mix, to provide a theoretical generalisation that would be transferable to other EDs.
5. As direct quotes are not presented, the reader does not have access to the actual quotes of the participants. However, we do not believe that the essential meaning has been significantly altered.

## 5 Conclusion

This is the first time that the concept of patient and family work has been applied to the ED journey, seeking to connect and contribute to the existing sociology of work, building on the earlier research of those who have previously described the patient experience. This study developed a typology of the work undertaken by the patients and their family, and how that related to the work of staff, and provided explanations for sensemaking gaps. Such a typology provides an alternative representation of the patient and family journey that can inform how staff might work differently to make patient and family sensemaking work more effective.

## Author Contributions

**Michael Clancy:** conceptualisation, data curation, investigation, methodology, project administration, original draft preparation, writing – review and editing. **Mark J. Johnson:** conceptualisation, writing – review and editing. **Joanne Turnbull:** conceptualisation, writing – review and editing.

## Funding

The authors received no specific funding for this work.

## Ethics Statement

Ethical approval was provided for this study by the Health Research Authority (18/WM/0358) and approved by the hospital research unit and the University of Southampton (ERGO 46904).

## Consent

All participants provided informed consent to participate in the study.

## Conflicts of Interest

The authors declare no conflicts of interest.

## Data Availability

The data that support the findings of this study are openly available at the University of Southampton Repository at http//eprints.soton.ac.uk/eprint, local eprint ID: 488847.
